# ANXIETY AND MEMORY COMPLAINTS IN MIDDLE-AGED AND OLDER ADULTS: THE MODERATING ROLE OF PHYSICAL ACTIVITY

**DOI:** 10.1093/geroni/igad104.1872

**Published:** 2023-12-21

**Authors:** Amy Costa, Ashley Curtis

**Affiliations:** University of South Florida, Tampa, Florida, United States; University of South Florida, Tampa, Florida, United States

## Abstract

The relationship between anxiety and memory complaints is well established. Additionally, physical activity has been shown to improve anxiety symptoms and cognition. Little research, however, has examined if physical activity impacts associations between anxiety and memory complaints in cognitively healthy middle-aged and older adults. Given memory complaints are often the first indicator of subsequent cognitive decline, it is important to understand factors that impact complaints. The present study tested whether physical activity moderated associations between anxiety and memory complaints. Adults aged 50+ (N=271, Mage=64.5, SD=7.8, 124 women) completed the International Physical Activity Questionnaire, Hospital Anxiety and Depression scale (HADS), and the Cognitive Failures questionnaire (CFQ). Multiple linear regressions and simple slope analyses were conducted to examine if physical activity moderated associations between anxiety and memory complaints (CFQ-memory), covarying for age, sex, income, difficulty walking, number of medical conditions, number of medications, body mass index, and depressive symptoms (HADS-depression). Results revealed total physical activity (R2-change=.01, p=.04), vigorous activity (R2-change=.02, p=.02), and walking activity (R2-change=.01, p=.03) moderated associations between anxiety and memory complaints. Specifically, higher anxiety was associated with more memory complaints at all levels of physical activity, with the magnitude of the effect largest at highest levels of physical activity. The present findings suggest higher amounts of physical activity may exacerbate the relationship between anxiety and memory complaints. It is possible aging adults with greater perceived memory disruption experience more anxiety and engage in health enhancing behaviors, such as physical activity. Future studies should explore temporal patterns of this relationship.

